# Campbell title registrations to date – October 2021

**DOI:** 10.1002/cl2.1198

**Published:** 2021-12-17

Details of new titles for systematic reviews or evidence and gap maps that have been accepted by the Editor of a Campbell Coordinating Group are published in each issue of the journal. If you would like to receive a copy of the approved title registration form, please send an email to the Managing Editor of the relevant Coordinating Group.

## CRIME AND JUSTICE

Interventions that address domestic abuse for mothers in or exiting prison: A systematic review

Michaela Rogers, Kelly Lockwood, Elizabeth Speake

CJCG

28 August 2021

Policing interventions to engage with immigrant communities: A systematic review

Michelle Sydes, Angela Higginson, Lorelei Hine, Lorraine Mazerolle

14 October 2021

## DISABILITY

Instruments for the evaluation of emotional intelligence in persons with hearing impairments: A scoping review

Petra Potmesilova, Milon Potmesil, Jana Mareckova

5 August 2021

## EDUCATION

The role of school‐work experience programmes in promoting career orientation in high school students: A systematic review

Donatella Poliandri, Mauro Palumbo, Antonio Fasanella, Alessandra Decataldo, Rita Marzoli, Sebastiano Benasso, Maria Dentale, Maria Paola Faggiano, Ughetta Favazzi, Brunella Fiore, Paola Giannoni, Grazia Graziosi, Monica Guerra, Veronica Lo Presti, Lorenzo Mancini, Beba Molinari, Enrico Nerli‐Ballati, Noemi Novello, Valentina Pacetti, Valeria Pandolfini, Fiorenzo Parziale, Stefania Sette, Claudio Torrigiani

24 August 2021

## INTERNATIONAL DEVELOPMENT

How does education affect health: A systematic reviewFatima Zahra, Nicole Haberland, Lauren Woyczynski, Stephanie Psaki

19 March 2021

Barriers and facilitators to enhancing access to business information through uptake of ICTs by women owning enterprises in low‐ and middle‐income countries: A qualitative synthesis

Ruth Nsibirano, Alison Annet Kinengyere, Agatha Kisa, Consolata Kabonesa, Howard White

30 April 2021

Value chain interventions for improving women's economic empowerment: A mixed‐method systematic review

Sabina Singh, Suchi Malhotra, Ashrita Saran, Howard White, Ranjitha Puskur, Hugh Sharma Waddington, Edoardo Masset

23 August 2021

The impact of agricultural mechanisation on women's economic empowerment: A mixed‐methods systematic review

Edoardo Masset, Monisha Narayan, Ashwani Verma, Ashrita Saran, Howard White, Ranjitha Puskur, Niyati Singaraju, Hugh Sharma Waddington

23 August 2021

## SOCIAL WELFARE

Global elder abuse: A mega‐map of systematic reviews on prevalence, consequences, risk and protective factors and interventions

Christopher Mikton, Yongjie Yon, Marie Beaulieu; Kevin St‐Martin, Julien Cadieux Genesse, Jennifer Storey, Fiona Campbell, Michaela Rogers, Amanda Phelan, Mark Byrne, Parveen Ali, David Burnes, Bridget Penhale, Tova Band‐Winterstein, Mark Lachs, Karl Pillemer, Lilly Estenson, Kelly Marnfelt

30 June 2021

Indigenous people's experiences and related processes that support healing and recovery from child sexual abuse: A systematic mapping literature review and evidence gap map

Graham Gee, Jordan Gibbs, Stephanie Brown, Helen Milroy

6 August 2021

Child and adolescent mental health and psychosocial support interventions: An evidence and gap map of low‐ and middle‐income countries

Manasi Sharma, Camila Perera, Alessandra Ipince, Shivit Bakrania, Farhad Shokraneh, Priscilla Idele, David Anthony, Prerna Banati

19 August 2021

Group‐based interventions for posttraumatic stress disorder: A systematic review and meta‐analysis of the role of trauma type

Siobhán M. Griffin, Elayne Ahern, Daragh Bradshaw, Orla T. Muldoon, Alastair Nightingale, Grace McMahon, Islam Bornica

21 August 2021

Informal social support interventions for improving outcomes for victim‐survivors of domestic violence and abuse: An evidence gap map

Karen Schucan Bird, Kate Hinds, Nicola Stokes, Carol Rivas, Martha Tomlinson

9 September 2021

